# A late-stage multi-dimensional intervention rescues lethal viral pneumonia in an aged hamster model

**DOI:** 10.1186/s43556-026-00521-5

**Published:** 2026-09-02

**Authors:** Ming Zhou, Xuan Liu, Jinxin Miao, Yanzhen Lin, Haiqing Xiao, Kun Wu, Longmei Zhang, Xinglin Li, Xinyi Yang, Lin Chen, Jian Ma, Wei Wang, Nan Xu, Jiang Li, Huachen Zhu, Yi Guan, Ningshao Xia, Tong Cheng, Lunzhi Yuan

**Affiliations:** 1 Present Address: State Key Laboratory of Vaccines for Infectious Diseases, Xiang An Biomedicine Laboratory, School of Life Sciences & School of Public Health, National Institute of Diagnostics and Vaccine Development in Infectious Diseases, Xiamen University, Xiamen, China; 2 Present Address: Clinical Center for Biotherapy, Clinical Research Center for Precision Medicine of Abdominal Tumor of Fujian Province, Xiamen Key Laboratory of Biotherapy, Zhongshan Hospital (Xiamen), Fudan University, Xiamen, China; 3 Present Address: Academy of Chinese Medicine Science, Henan University of Chinese Medicine, Zhengzhou, China; 4 Present Address: Department of Obstetrics and Gynecology, Zhongshan Hospital of Xiamen University, Xiamen, China; 5 Department of Infectious Diseases, First Affiliated Hospital of Anhui Medical University, Hefei, China; 6 State Key Laboratory of Emerging Infectious Diseases, School of Public Health, Li Ka Shing, Faculty of Medicine, The University of Hong Kong, Hong Kong SAR, China; 7 Guangdong-Hong Kong Joint Laboratory of Emerging Infectious Diseases, Joint Institute of Virology (STU/HKU), Shantou University, Shantou, China; 8 Shanghai Institute of Virology, Shanghai-Jiao-Tong University School of Medicine, Shanghai, China

**Keywords:** Respiratory virus infections, Fatal severe pneumonia, Aging, SARS-CoV-2, Disease enhancement, Combined therapy

## Abstract

Severe pneumonia caused by respiratory virus infection is one of the main threats to the lifespan of the aged population and physiological aging fundamentally drives poor disease outcomes in respiratory viral infections, necessitating investigation of the underlying mechanisms and the development of effective countermeasures. Pulmonary transcriptomic profiling reveals that diffuse cell death, inadequate antiviral responses, myeloid-driven excessive inflammation and immune-thrombosis dictate the pulmonary microenvironment are typical molecular pathology characteristics of the SARS-CoV-2-infected aged hamsters rather than the adult controls. The elevated pathological baseline and dysregulated immune responses are demonstrated as the key host factors of lethal viral pneumonia in the aged hamsters. Meanwhile, SARS-CoV-2 infection in the adult hamsters usually resulted in an aged-like pulmonary transcriptomic signature, suggesting a potential link among aging, dysregulated immune responses and severe illness. To reverse the progression of lethal severe pneumonia in the aged hamsters, we initiated a multidimensional combination therapy of dexamethasone, heparin and the broad-spectrum viral decoy CoVR-MV at three days after infection. Although single- and dual-drug therapies were insufficient to achieve functional cure, the three-drug combination therapy resulted in a potent reduction of high mortality, body weight loss, viral load, lung pathology and cytokine storm through the synergism of immunoregulatory, anticoagulant and antiviral effects, effectively intercepting the multifaceted pathogenic network. This study highlights physiological aging as a core driver of critical COVID-19 pathogenesis and provides valuable clues for the rational design of multi-drug and multi-target therapies against respiratory viral infections and lethal severe pneumonia in the elderly population.

**Supplementary Information:**

The online version contains supplementary material available at https://doi.org/10.1186/s43556-026-00521-5.

## Introduction

The burden of infectious diseases increasing with aging has become a critical global public health problem. Clinically, respiratory virus infections are one of the major threats to the life expectancy and health in the elderly population [1–4]. In recent decades, pandemic and seasonal epidemics of respiratory viruses, including influenza virus (IFV), coronavirus (CoV) and respiratory syncytial virus (RSV), have caused millions of deaths. Numerous studies have shown that the elderly population has a higher mortality, severity and hospitalization rate than adult following infection with CoV, IFV and RSV. The mortality rate of IFV infection increases exponentially with age, surging from a baseline of ≤ 6.4 per 100,000 individuals under 65 to a peak of up to 223.5 per 100,000 in those aged 75 and older [5]. Similarly, while RSV infection is largely asymptomatic in adult populations, it leads to high intensive care unit (ICU) admission rates in the elderly, with a mortality rate ranging from 6% to 8% among hospitalized patients [6]. Furthermore, the SARS-CoV-2 pandemic has starkly highlighted this age-dependent vulnerability, with mortality rates skyrocketing from a mere 0.32% in individuals under 60 to an alarming 13.4% in patients aged 80 and older [7]. In addition, rather than exhibiting typical acute-phase symptoms such as fever and cough, aged individuals frequently present with atypical manifestations of coronavirus disease 2019 (COVID-19) such as delirium and lethargy [8, 9]. These insidious nature of early symptoms might defer clinical intervention to an advanced stage. Frequently, the critical cases of respiratory viral infections in aged hosts are characterized by high viral burdens, severe pneumonia, cytokine storms, coagulopathy, multi-organ failure, and death [10, 11].

However, current medicines cannot fully meet the growing need for urgent treatment of respiratory viral infections and fatal severe pneumonia, especially in elderly people with critical COVID-19 [12]. Although many antiviral drugs against SARS-CoV-2 have been approved or licensed for emergency use, drugs to relieve immunological and pathological manifestations are still lacking [13]. Another challenge is the timing of treatment intervention. Most of the approved drugs or clinical treatment protocols have a good therapeutic effect in early intervention, but a significant decrease has been observed in late intervention. For example, patients treated with tolizumab in the early phase of COVID-19 had a lower risk of in-hospital death than those treated with tolizumab in the mid to late phase [14]. In the critical COVID-19 patients, early anti-inflammatory treatment with intravenous anakinra resulted in a 14% mortality rate, which increased to 43% with late anakinra treatment [15]. Similarly, remdesivir failed to show a therapeutic advantage in patients with severe disease, showing a higher mortality rate compared to the control group [16]. Therefore, understanding the underlying mechanisms of aging and disease exacerbation and developing effective countermeasures are essential to reduce the risk of respiratory viral infections in the elderly population.

It has been reported that aging promotes immune cell senescence, inflammation and decline in physiological function, which are predisposing risk factors for respiratory virus infection [17]. On the other hand, virus infection usually leads to premature aging of the respiratory and immune systems [18], which may undermine the protective effect of the vaccine and host resistance to reinfection. To interpret the potential interaction between physiological aging and respiratory viral infection, we used a highly simulated COVID-19 animal model of the Syrian hamster. In previous studies by us and others, the hamster model has been widely used to measure the infectivity, transmissibility and pathogenicity of different SARS-CoV-2 variants [19–24], as well as the efficacy of drugs and vaccines [25–32].

In this study, we compared the disease outcomes of SARS-CoV-2 infection in 10–20-week-old adult and 80–90-week-old hamsters and performed transcriptome analysis in lung tissue. Following SARS-CoV-2 infection, transcriptome features such as diffuse cell death, inadequate antiviral responses, excessive inflammation and coagulation abnormalities were observed in the aged hamsters, similar to those observed in the critical COVID-19 patients. Such a multifaceted pathogenic network might be one of the key factors behind the clinical failure of current monotherapies in aged hosts. Based on this, we set out to design a combined immunoregulatory, anticoagulant and antiviral therapy strategy using the anti-inflammatory drug dexamethasone (Dex), the anticoagulant drug heparin (Hep) and the bio-mimic cellular vesicle coronavirus decoy CoVR-MV. By systematically evaluating the survival rate, body weight loss, degree of lung injury, respiratory viral load, and lung inflammatory cytokine levels in SARS-CoV-2-infected aged hamsters, we demonstrated that the triple-drug regimen strategy provides superior therapeutic efficacy over single- and double-drug therapies. Ultimately, these findings highlight a highly translatable, multi-target paradigm for treating lethal severe pneumonia in the elderly population.

## Results

### Aging exacerbates disease severity in hamsters across prototype and variant SARS-CoV-2 infections

**Fig. 1:**
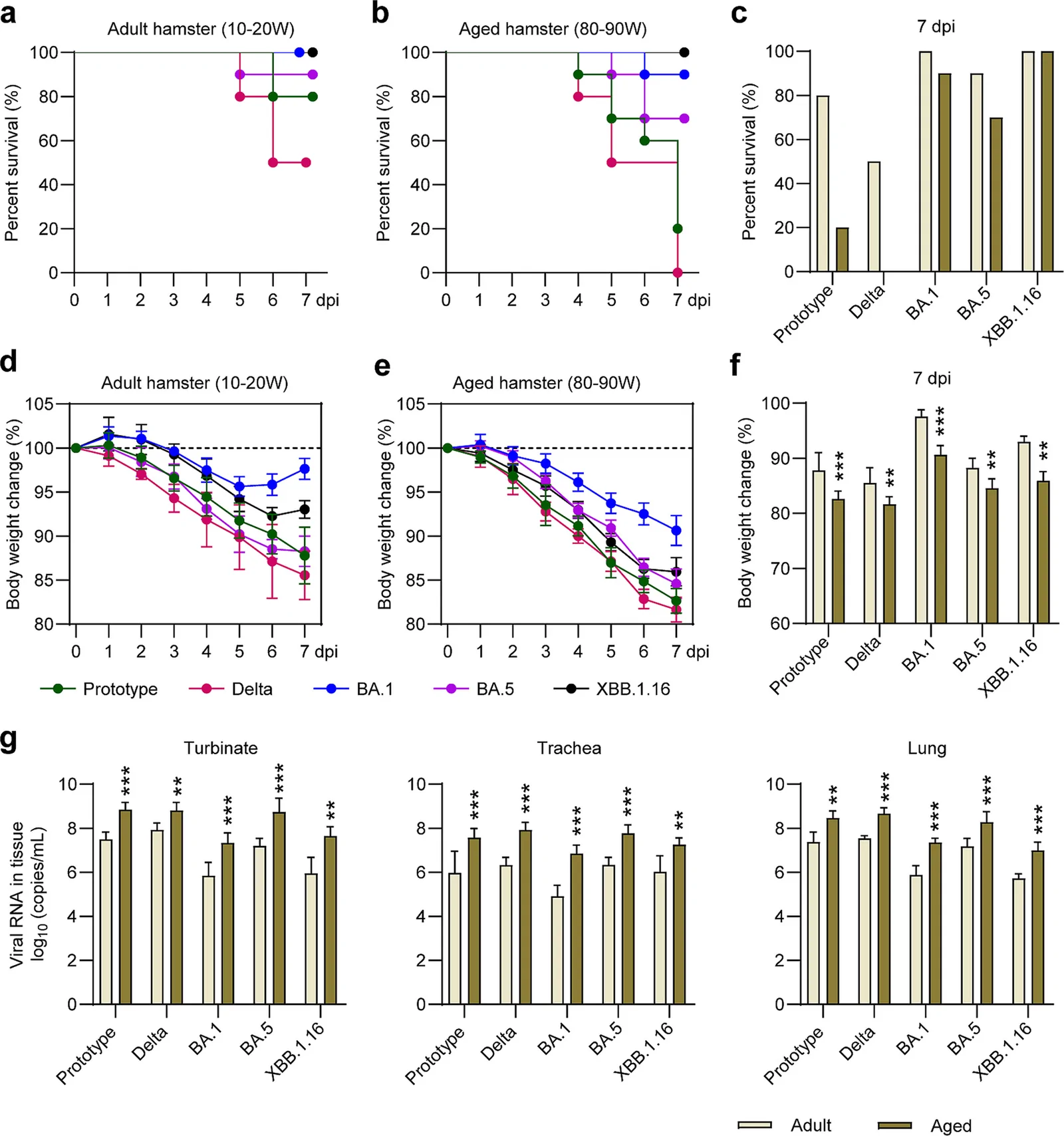
Disease outcomes of SARS-CoV-2 infection in adult and aged hamsters. Adult (10-to-20 week old) and aged (80-to-90 week old) hamsters after intranasally inoculated with 1 × 10^4^ PFU of different SARS-CoV-2 variants, including the prototype, Delta, Omicron BA.1, BA.5 and XBB.1.16, respectively. **a**-**b** Survival rates of these hamsters (n = 10) and **d**-**e** body weight changes (n = 4) of the survived hamsters were recorded within seven days. In each group, the **c** survival rate and **f** body weight loss of adult and aged hamsters at 7 dpi were shown, respectively. **g** Nasal turbinate, trachea, and lung tissues of the hamsters were collected at 7 dpi. Viral RNA levels in tissue homogenates were measured by qRT-PCR, respectively (n = 4). Primers targeting the SARS-CoV-2 ORF1ab gene were used for amplification. Significance was determined by one-way ANOVA and shown in heat map. Two-sided P values < 0.01 were considered significant: *, *P* < 0.01; **, *P* < 0.001; ***, *P* < 0.0001

To validate the age-dependent disease enhancement of respiratory virus infection, adult and aged hamsters were infected with 1 × 10^4^ PFU of different SARS-CoV-2 strains, including prototype, Delta, Omicron BA.1, BA.5 and XBB.1.16. To assess disease outcome, we recorded the survival rates and body weight changes of these hamsters from 0 to 7 days post infection (dpi). All adult hamsters survived after infection with Omicron BA.1 and XBB.1.16 (Fig. 1a). Meanwhile, the survival rates of adult hamsters infected with Prototype, Delta and BA.5 were 80%, 50% and 90%, respectively (Fig. 1a). However, the survival rates of the aged hamsters infected with prototype, Delta, Omicron BA.1, BA.5 and XBB.1.16 decreased to 20%, 0%, 90%, 70% and 100%, respectively (Fig. 1b). The aged hamsters showed higher mortality than the adult controls at 7 dpi (Fig. 1c). The adult hamsters showed body weight loss of 12.2 ± 3.2%, 14.5 ± 2.8%, 2.4 ± 1.2%, 11.7 ± 1.7% and 6.9 ± 1.0% at 7 dpi of prototype, Delta, Omicron BA.1, BA.5 and XBB.1.16 infection, respectively (Fig. 1d). The aged hamsters showed a body weight loss of 17.3 ± 1.4%, 18.4 ± 1.4%, 9.3 ± 1.8%, 15.6 ± 1.7% and 14.1 ± 1.4% at 7 dpi of Prototype, Delta, Omicron BA.1, BA.5 and XBB.1.16 infections, respectively (Fig. 1e). The aged hamsters showed more body weight loss than the adult controls at 7 dpi (Fig. 1f). To evaluate viral replication, we measured SARS-CoV-2 RNA loads in the upper and lower respiratory tracts (turbinate, trachea, and lung) at 7 dpi by quantitative reverse transcription polymerase chain reaction (qRT-PCR). In all of the tested SARS-CoV-2 strains, the aged hamsters showed significantly higher viral RNA levels than the adult controls in respiratory tract organs including the turbinate, trachea and lung (Fig. 1g). Moreover, the aged hamsters that survived infections with the BA.1, BA.5, and XBB.1.16 variants showed more than tenfold lower serum neutralizing antibody (NAb) titers and slower body weight recovery than the adult controls from 14 to 28 dpi (Fig. S1). The above data showed that aged hamsters had a poorer disease outcome and prognosis than adult hamsters after infection with all five SARS-CoV-2 strains tested, suggesting an age-dependent disease enhancement.

### Transcriptomic profiling reveals age-dependent hyper-inflammation and pro-thrombotic signatures post-infection

**Fig. 2:**
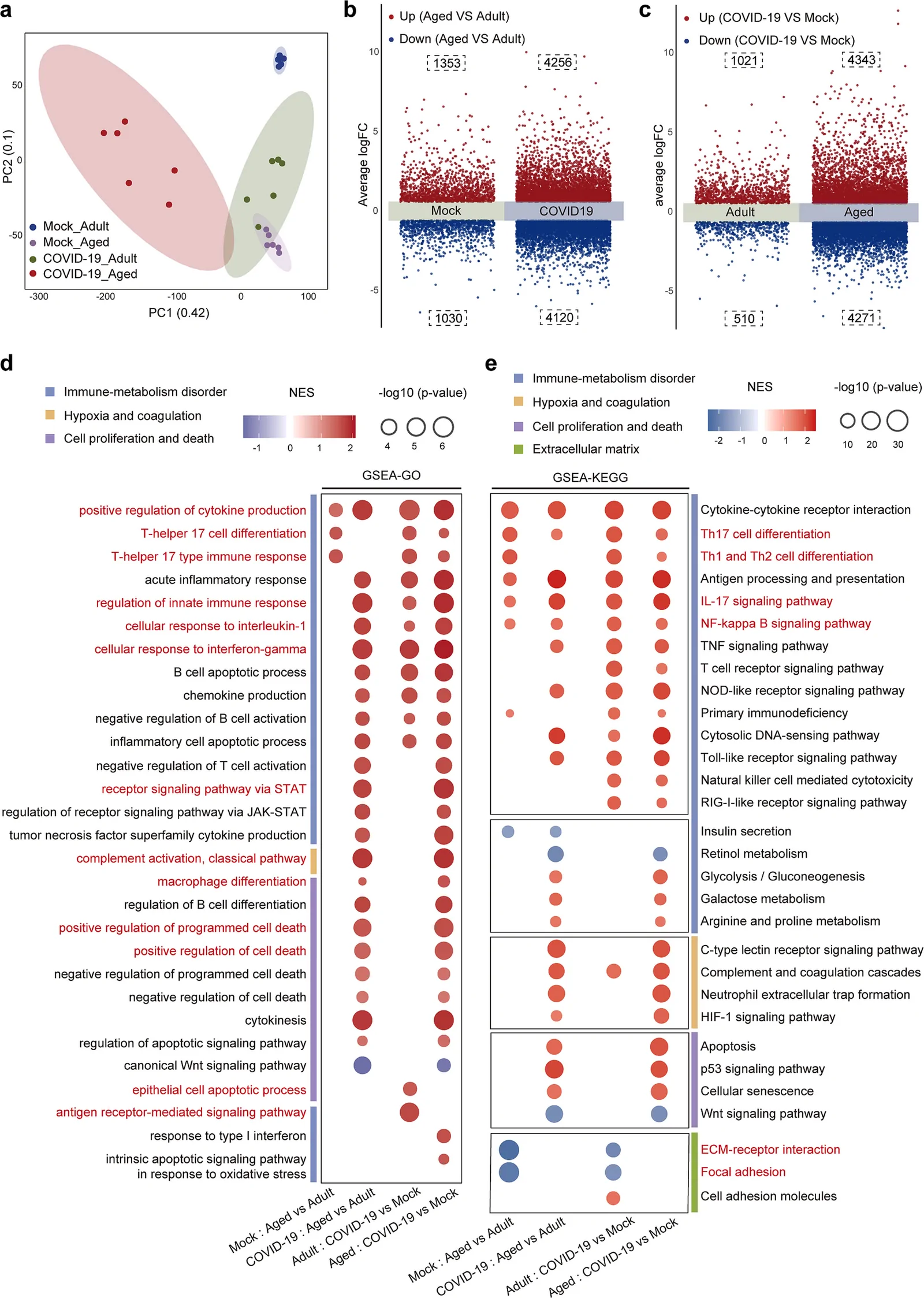
Differentiation in transcriptomic alterations in the lung tissues of adult and aged hamsters with or without SARS-CoV-2 infections. **a** Principal component analysis of the mock_adult (n = 6), mock_aged (n = 6), COVID-19_adult (n = 6) and COVID-19_aged hamsters (n = 6). Volcano plot distribution for each group was shown according to transcriptomic data. **b** Volcano plots comparing transcriptomes of aged hamsters with adult controls in mock or COVID-19 hamsters. **c** Volcano plots comparing transcriptomes of COVID-19 with mock controls in adult or aged hamster. The results of the GSEA using **d** GO or **e** KEGG as gene sets. The color intensity represents the Normalized Enrichment Score (NES) calculated by the GSEA for these modules. Dot size presents p value, with only p < 0.05 depicted here

To delineate the underlying pathology mechanisms of age-dependent disease enhancement after SARS-CoV-2 infection (Delta), we analyzed the transcriptome differences in lung tissues collected from adult and aged hamsters at 5 dpi. Lung tissues collected from age-paired mock hamsters without infection were set as controls. The results of principal component analysis (PCA) of gene expression showed that hamsters within the same group were closer together, with good differentiation between the groups and no outliers produced (Fig. 2a). The trends of transcriptome features after infection in aged hamsters are not consistent with those of adult hamsters. Interestingly, the mock-aged and COVID19-adult hamsters showed similar trends of transcriptome features, suggesting SARS-CoV-2 infection might accelerate aging-related pulmonary remodeling (Fig. 2a). The diverse gene expressions in the four hamster groups were further analyzed for differences and shown by volcano diagrams (Fig. 2b, c). Before SARS-CoV-2 infection, aged hamsters showed up-regulation of 1,353 genes and down-regulation of 1,030 genes than adult hamsters. After SARS-CoV-2 infection, aged hamsters showed up-regulation of 4,256 genes and down-regulation of 4,120 genes than adult hamsters, adult hamsters showed up-regulation of 1,021 genes and down-regulation of 510 genes than age-paired mock hamsters, aged hamsters showed up-regulation of 4,343 genes and down-regulation of 4,271 genes than age-paired mock hamsters, respectively. Of note, SARS-CoV-2 infection in aged hamsters results in more significant changes of genes in lung tissues than adult hamsters.

Afterwards, we performed Gene Set Enrichment Analysis (GSEA) enrichment analysis based on the Gene Ontology (GO) database (Fig. 2d). Before SARS-CoV-2 infection, the aged hamsters showed up-regulation of cytokine production, Th17 cell differentiation and response associated signaling pathways and genes than the adult hamsters, which indicated a higher inflammation baseline (Fig. 2d). After SARS-CoV-2 infection, more significant up-regulation of acute inflammatory responses such as interferon gamma (IFN-γ, a critical cytokine for innate immunity), interleukin-1 (IL-1, a potent pro-inflammatory mediator) and JAK-STAT (a major immune signaling pathway), complement activation and programmed cell death-associated signaling pathways and genes were observed in the aged hamsters rather than the adult hamsters (Fig. 2d). Of note, disturbance of immune responses after SARS-CoV-2 infection was more extensive and deeper in the aged hamsters. For instance, type I interferon-mediated innate immune responses, B-cell and macrophage differentiation-associated, complement-, and programmed cell death-associated signaling pathways and genes were up-regulated only in the aged hamsters after SARS-CoV-2 infection (Fig. 2d). In contrast, antigen receptors and epithelial apoptosis-related signaling pathways and genes are up-regulated only in the adult hamsters after SARS-CoV-2 infection (Fig. 2d). These data suggest that the adult and aged hamsters display diverse patterns of immune responses and immune-pathophysiological changes after SARS-CoV-2 infection.

Furthermore, Kyoto Encyclopedia of Genes and Genomes (KEGG) pathway analysis revealed that varied signaling pathways and genes were mainly enriched in immune-metabolism disorder, coagulation and hypoxia, cell growth and death, and cell matrix (Fig. 2e). In the mock hamsters, aging was related to up-regulation of Th1, Th2, Th17 cell differentiation, IL-17 and NFκB, as well as down-regulation of insulin secretion, ECM receptor interactions and Focal adhesion associated signaling pathways and genes (Fig. 2e), which indicated a higher inflammation baseline and dysfunction of lung tissue. After SARS-CoV-2 infection, the aged hamsters showed complicated immune-metabolism disorders, significant up-regulation of coagulation and hypoxia, programmed cell senescence and death-associated signaling pathways and genes (Fig. 2e). In the adult and aged hamsters, SARS-CoV-2 infection resulted in up-regulation of innate immune response and acute inflammatory responses (Fig. 2e). For instance, complement-related genes such as complement C5a receptor 1 (C5ar1) and type I interferon pathway-related genes such as Stat2 and Sting1 were up-regulated after SARS-CoV-2 infection in both adult and aged hamsters (Fig. S2). Signaling pathways and genes of metabolism, coagulation and hypoxia, cell growth and death were not significantly changed in the adult hamsters with SARS-CoV-2 infection (Fig. 2e). In contrast, significant up-regulation of complement and coagulation cascades, neutrophil extracellular trap formation, HIF-1 signaling pathway was caused by SARS-CoV-2 infection in the aged hamsters, as well as activation of apoptosis and cellular senescence (Fig. 2e). For instance, representative inflammatory cytokines such as IL-6, hypoxia associated genes such as HIF-1a, and complement activation associated genes such as C1r, C1s, and C8a were up-regulated only after SARS-CoV-2 infection in the aged hamsters (Fig. S2). Combining the physiology and virology data, transcriptome analysis of hamster lung tissues indicated that inadequate innate immune responses, high viral load, excessive inflammatory responses, cell death and tissue injury, over-activation of complement and coagulation cascades (abnormal coagulation) are the representative characteristics and important aggravating factors for the poor disease outcome of SARS-CoV-2 infection in the aged hamsters.

### Aged hamsters with critical COVID-19 exhibit maladaptive immune reprogramming driven by hyper-inflammation and cytotoxicity

To delineate the remodeling of the pulmonary immune microenvironment in the aged hamsters, we applied the CIBERSORT algorithm to perform transcriptomic deconvolution of the bulk RNA-seq data. For the compartment of myeloid immune cells, the uninfected adult and aged hamsters exhibited comparable baseline profiles. Following SARS-CoV-2 infection, however, the aged hamsters displayed a significant decline in the proportion of activated natural killer (NK) cells, which are responsible for viral clearance. Furthermore, the aged hamsters exhibited a massive expansion of highly pro-inflammatory M1 macrophages, while local monocyte and neutrophil populations underwent further severe depletion from an already lower baseline (Fig. S3a). For the compartment of lymphoid immune cells, CD4^+^ T helper cell 1 (Th1), CD4^+^ T follicular helper cells (Tfh) and memory CD8^+^ T cells (Tmem) were increased in the aged hamsters after infection (Fig. S3b). The significant decrease of Naïve CD4^+^ T cells indicated that SARS-CoV-2 infection causes immune exhaustion phenotypes in the aged hamsters (Fig. S3b). Because of the lack of antibodies specific to the markers of hamster immune cells, we only validated the changes of pulmonary M1 macrophages, Th1, Tfh and Naïve CD4^+^ T cells by fluorescence-activated cell sorting (FACS). The proportions of these immune cell subsets showed similar trends in the results of both RNA-Seq and FACS analysis. M1 macrophages, Th1 and Tfh were increased after SARS-CoV-2 infection, and the aged hamsters showed higher levels than the adult controls in M1 macrophages and Th1 (Fig. S4). Conversely, the proportions of Naïve CD4^+^ T cells were decreased after SARS-CoV-2 infection and the aged hamsters showed lower levels than the adult controls. Such an inverted cellular landscape implies that the aged immune system completely uncouples from coordinated antiviral defense, defaulting instead to an undirected pathological hyperactivation.

Afterwards, we quantified core effector molecules in the hamster lung tissues by qRT-PCR, which were highlighted in the results of transcriptome (Fig. S5). Firstly, the profound upregulation of key cytokines (*IL-6*, *TNF-α*, *IL-1β* and *CXCL10*) biochemically verified the hyper-inflammatory phenotype driven by M1 macrophage polarization. Meanwhile, the observed depletion of local neutrophils was accompanied by an elevated expression of core NETosis-driving enzymes (*MPO* and *ELANE*). The results indicate that such cellular loss originates from extensive extracellular trap formation rather than simple apoptosis, thereby directly fueling local micro-thrombosis. Moreover, the aberrant accumulation of CD8^+^ memory T cells translated into severe bystander cytotoxicity, as evidenced by the pathological overexpression of cytotoxic granules (*IFN-γ* and *GZMB*) in the aged lung tissues. Furthermore, this immune-mediated damage precipitated severe endothelial injury and a hypercoagulable state, which was explicitly validated by the massive upregulation of coagulation cascade initiators and fibrinolysis inhibitors (*SERPINE1*). Collectively, the integration of cellular and molecular profiles reveals that the aged pulmonary microenvironment undergoes a profound maladaptive reprogramming, characterized by the collapse of targeted viral clearance and a compensatory, highly destructive hyper-inflammatory network.

### Transcriptomic alterations revealed the similarity between aging process and SARS-CoV-2 infection in hamster model

**Fig. 3:**
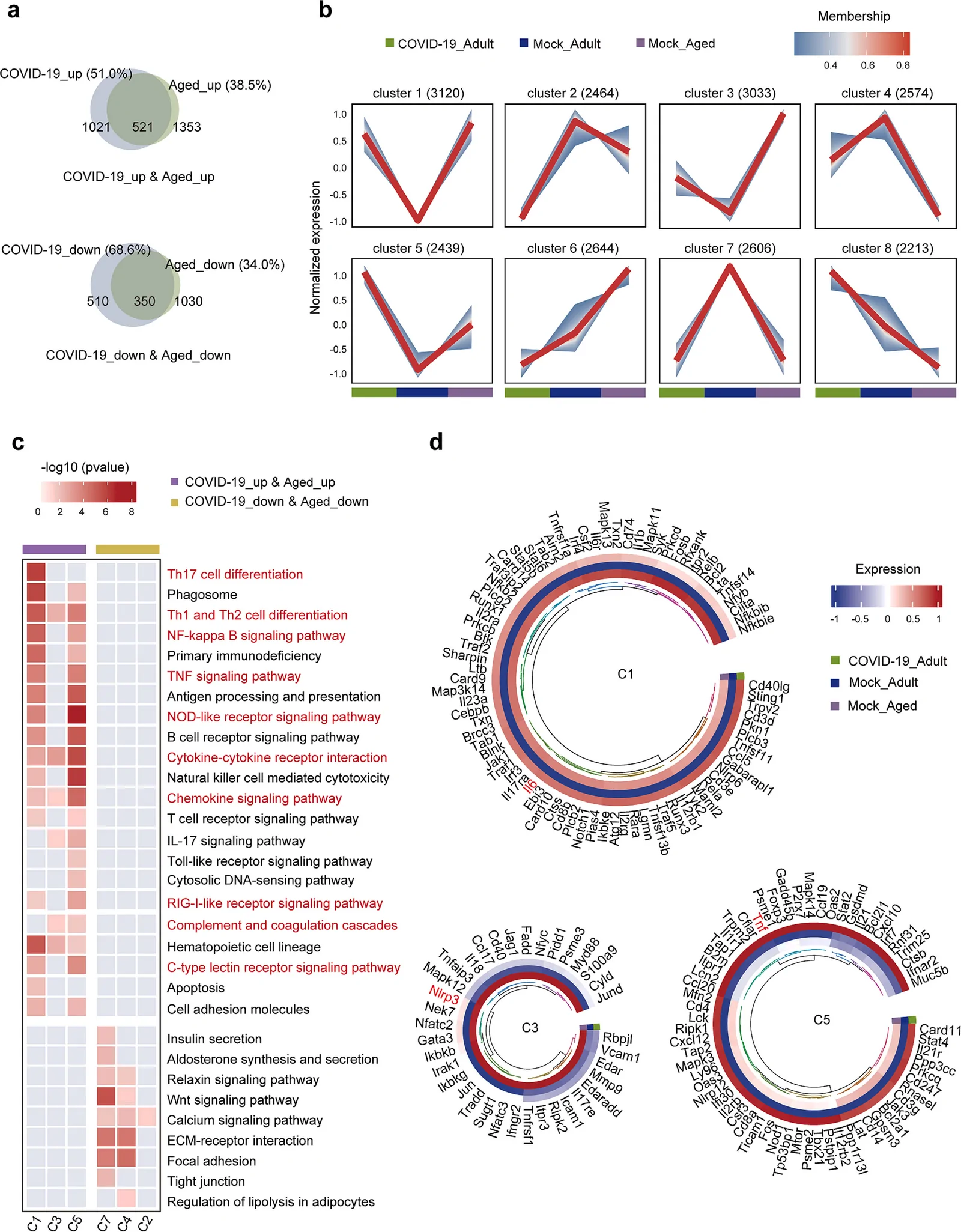
Transcriptomic alterations revealed the similarity between the aging process and SARS-CoV-2 infection. **a** The pie chart displays the number of genes that can be upregulated or downregulated by both SARS-CoV-2 infection and the aging process in the mock hamsters. **b** Transcriptomes in mock_adult, mock_aged, COVID-19_aged and COVID-19_adult hamsters were clustered with mfuzz into eight discrete significant clusters. Red lines represent the average trajectory for each cluster. **c** KEGG pathway enrichment analysis of gene clusters commonly upregulated or downregulated by both physiological aging and SARS-CoV-2 infection. The color intensity is employed to indicate the p value of the path, with grey representing an insignificant change. **d** The expression of representative genes of clusters. The heatmap illustrates genes commonly upregulated by both SARS-CoV-2 infection and physiological aging relative to uninfected controls

As the PCA results showed the similarity between the aging process and SARS-CoV-2 infection (Fig. 2a), we employed Venn diagrams to display the intersection of differential genes between the mock aged hamsters and adult hamsters with or without SARS-CoV-2 infection. Generally, SARS-CoV-2 infection up-regulated 1021 genes in the lung of adult hamsters and 521 of them were also up-regulated in the mock aged hamsters (Fig. 3a). SARS-CoV-2 infection down-regulated 510 genes in the lung of adult hamsters and 350 of them were also down-regulated in the mock aged hamsters (Fig. 3a). For a more comprehensive understanding of the similarity between the aging process and SARS-CoV-2 infection, we clustered the expression of differential gene expression trends in the three groups for eight clusters by the Mfuzz algorithm (Fig. 3b). The genes in clusters 1, 3 and 5 were up-regulated by both aging process and SARS-CoV-2 infection, and genes in 2, 4 and 7 were both down-regulated. In contrast, aging process and SARS-CoV-2 infection resulted in contrary gene expression trends in the clusters 6 and 8. Pathway enrichment analysis further demonstrated that the signaling pathways of innate and adaptive immune perturbation, coagulation and apoptosis, such as T-cell differentiation, antigen processing and presentation, natural killer cell mediated cytotoxicity, NFκB, tumour necrosis factor (TNF, a critical pleiotropic cytokine regulating systemic inflammation), RIG-I and NOD-like receptor, C-type lectin receptor, complement and coagulation cascades, cytokines and chemokines are enriched and up-regulated in the clusters 1, 3 and 5 (Fig. 3c, left). On the other hand, the signaling pathways of physiological function and structure such as metabolism, cell growth, Wnt, relaxin, calcium channel, insulin secretion and extracellular matrix receptor interaction are enriched and down-regulated in clusters 2, 4 and 7 (Fig. 3c, right). Furthermore, the key varied genes in each cluster were displayed (Fig. 3d, Fig. S6). Remarkably, genes of representative inflammatory cytokines such as IL-6, NLRP3 (a core inflammasome sensor driving pyroptosis) and TNF were up-regulated in clusters 1, 3 and 5, respectively (Fig. 3d). In conclusion, the overlap of aging and viral infection leads to poor disease outcome, suggesting that physiological aging has dual disease attributes including mild basic disease and exacerbator of critical COVID-19.

### Late triple-drug therapy of Dex, Hep, and CoVR-MV abrogates mortality in aged hamsters with critical COVID-19

**Fig. 4:**
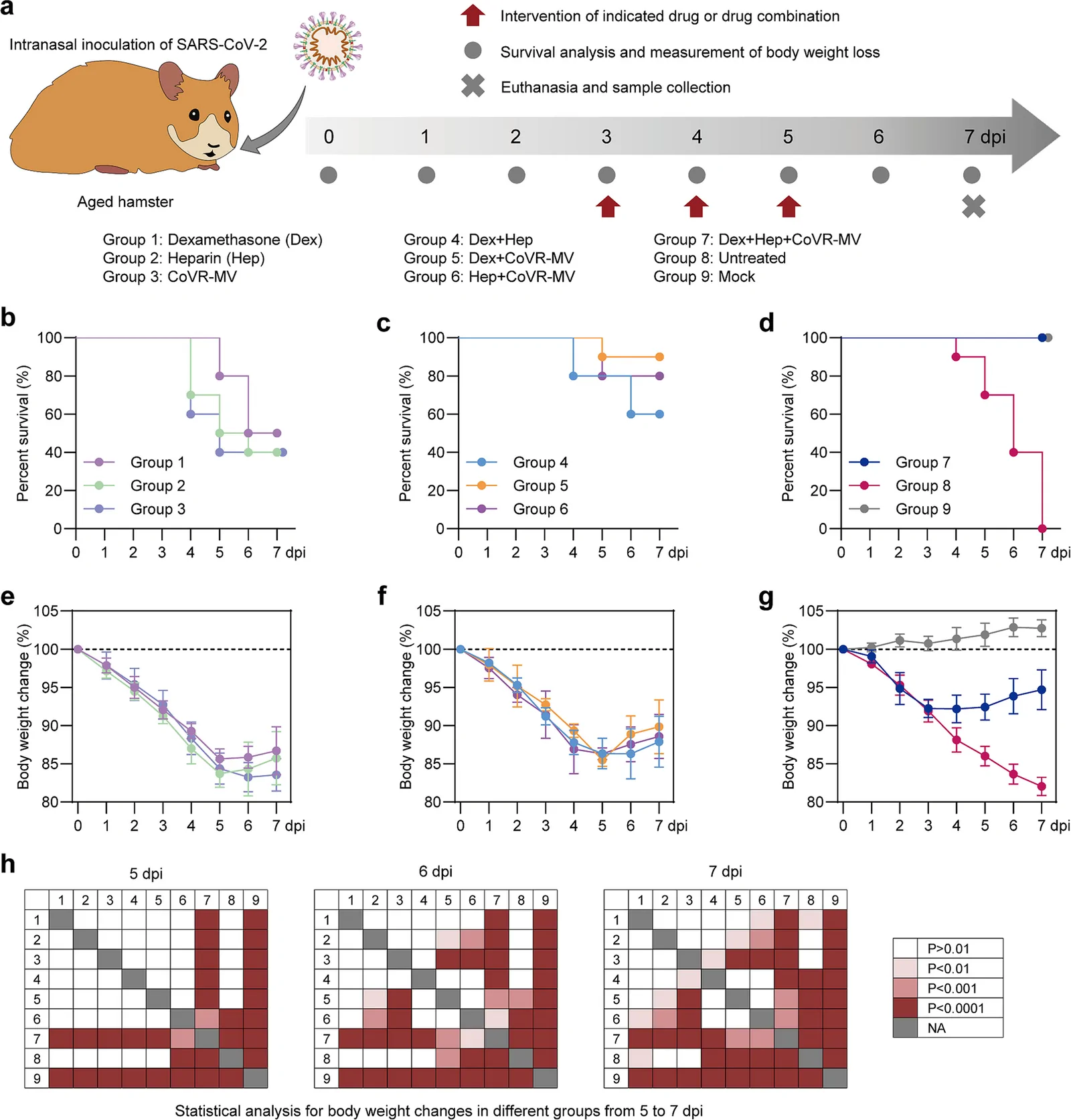
Physiological characteristics of aged hamsters after infection of SARS-CoV-2 and late intervention of different drugs. **a** The schematic diagram shows the process of virus inoculation, late intervention of different drugs and sampling from 0 to 7 dpi. **b-d** Survival rate is shown for the mock hamsters and the SARS-CoV-2-infected hamsters with or without therapy (n = 10). **e-g** Changes in body weight are shown from 0 to 7 dpi (n = 4). **h** Heat map statistical analysis of body weight data for each group from 5 to 7 dpi. Significance was calculated by two-way ANOVA. Two-sided P values < 0.01 were considered significant:* P* < 0.01 (Light red), *P* < 0.001 (Medium red), *P* < 0.0001 (Deep red); *P* > 0.01 indicates no significance (White)

Next, we investigated whether single use of anti-inflammatory agent Dex, anti-coagulant agent Hep, virus decoy CoVR-MV and their combinations would reserve critical COVID-19 in the aged hamster at a late intervention. To compare the therapeutic effects of these drugs and different combinations, 80-week-old male hamsters were intranasally inoculated with 1 × 10^4^ PFU of SARS-CoV-2 Delta variant. In the groups of single drug therapy, infected hamsters received three doses of Dex, Hep, or CoVR-MV at 3, 4, and 5 dpi (groups 1–3, respectively; Fig. 4a). Hamsters receiving dual-drug therapies (Dex and Hep, Dex and CoVR-MV, Hep and CoVR-MV) and the triple-drug combination were designated as groups 4–7, respectively. The untreated infected hamsters and uninfected mock hamsters served as the control cohorts (groups 8 and 9). Throughout the animal experiment, we monitored the survival rate of these aged hamsters from 7 dpi. In addition, a separate set of hamsters was used for detection of body weight loss and sacrificed at 7 dpi to evaluate the pathological, virological, and immunological characteristics of COVID-19 progression. The hamsters in groups 1 to 9 showed survival rates of 50%, 40%, 40%, 60%, 90%, 80%, 100%, 0% and 100% at 7 dpi, respectively (Fig. 4b to d). Notably, the hamsters in groups of combined therapy (groups 4 to 7) were more likely to survive from critical COVID-19. The hamsters in groups 1 to 9 showed body weight changes of 86.7 ± 3.1, 85.7 ± 3.5, 83.5 ± 2.1, 87.9 ± 3.3, 89.9 ± 3.5, 88.6 ± 2.9, 94.7 ± 2.6, 82.1 ± 1.2, and 102.8 ± 1.1 at 7 dpi, respectively (Fig. 4e to g). Statistical analysis of body weight changes suggested that hamsters with the combination of three-drug displayed significantly less body weight loss than the untreated hamsters and those with single-drug or two-drug therapy (Fig. 4h). In a parallel experiment, the survived hamsters in group 7 showed higher serum neutralization antibody titer levels and more rapid body weight recovery than those in groups 1–6 from 14 to 28 dpi, respectively (Fig. S7). In addition, late intervention of middle or low doses of single drug treatment or high dose Nirmatrelvir and Ritonavir treatment can rarely rescue the aged hamsters intranasally infected with 1 × 10^4^ PFU of Delta variant, highlighting that appropriate drug dosage and multi-drug combination strategy are both important to ensure the treatment effectiveness of lethal viral pneumonia (Fig. S8). Overall, these data demonstrat that combined therapy of Dex, Hep and CoVR-MV is an optimized option to rescue the SARS-CoV-2-infected aged hamsters from death and reduce sharp body weight loss.

### The triple therapy effectively resolves pulmonary immunopathology and viral burden

**Fig. 5:**
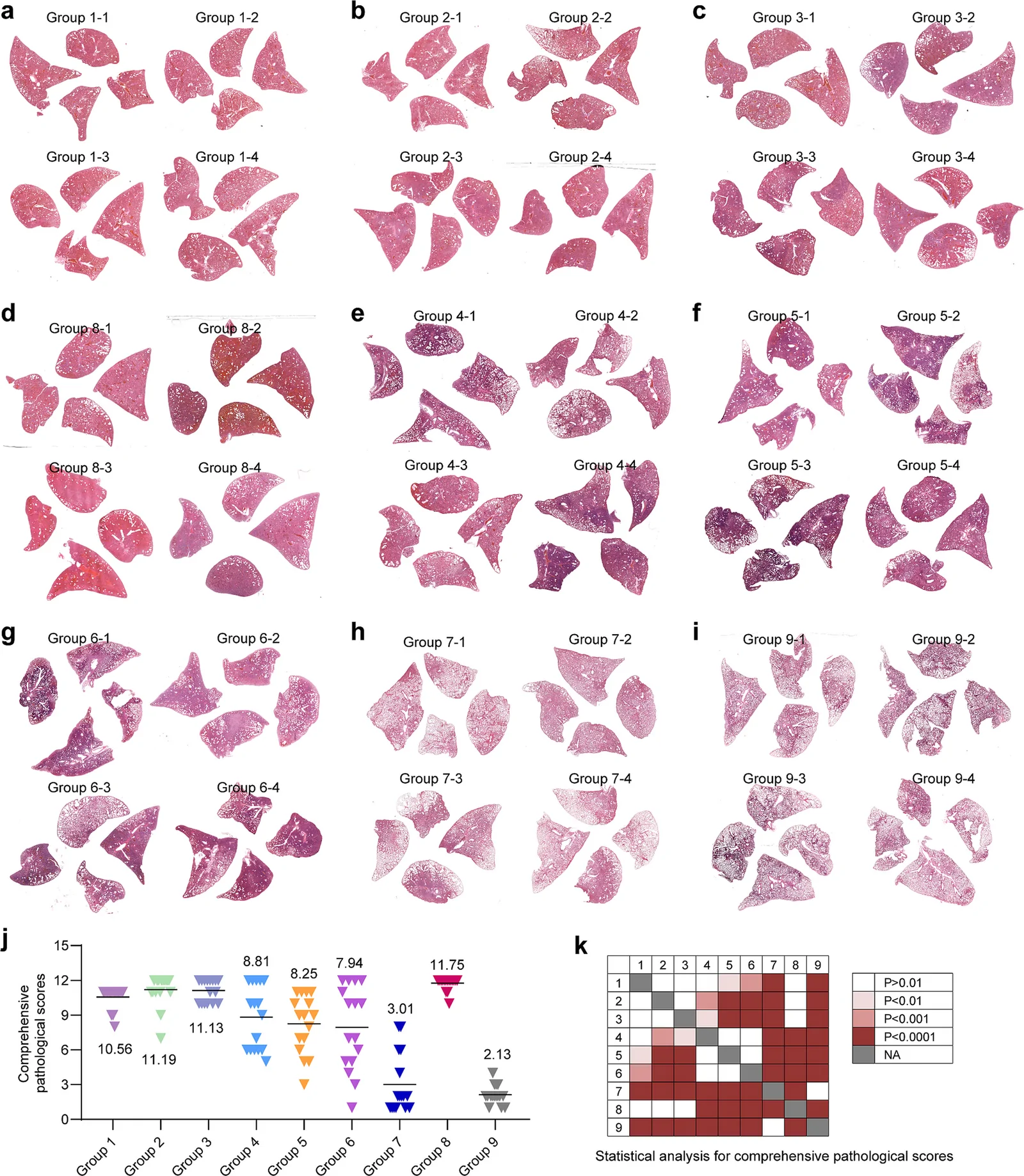
Pathological changes in lung lobes of the aged hamsters. **a**-**i** Overall screening for the H&E staining lung lobe sections of the hamsters in groups 1–9 that deceased at 7 dpi, respectively (n = 4). For each hamster, four out of the five lung lobes were shown. **j** Comprehensive pathological scores were assigned to lung sections based on the severity and percentage of injured areas in each lung lobe. Detailed information of the pathological scores was shown in Table S1. **k** Significance of the pathological scores was determined by one-way ANOVA and shown in heat map. Two-sided P values < 0.01 were considered significant: *P* < 0.01 (Light red), *P* < 0.001 (Medium red), *P* < 0.0001 (Deep red); *P* > 0.01 indicates no significance (White)

Furthermore, lung pathological changes, viral RNA load in respiratory tract organs and mRNA levels of proinflammatory cytokines in lung homogenates of the deceased hamsters and those euthanized at 7 dpi were analyzed. Hematoxylin and eosin (H&E) staining for lung lobes was performed to evaluate the degree of lung pathological changes. Representative features of severe pneumonia such as alveolar structural disintegration, haemorrhage and abnormal recruitment of immune cells were observed in all of the lung lobes collected from hamsters in groups 1–3 (Fig. 5a to c) and group 8 (Fig. 5d). In contrast, the severity of tissue damage was slightly reduced in part of the lung lobes of hamsters in groups 4–6 (Fig. 5e to g). Notably, the lung lobes collected from hamsters in group 7 showed a mild to moderate degree of tissue injury (Fig. 5h), which is more similar to the features of lung lobes collected from the mock hamsters (Fig. 5i). In addition, the severity of lung pathological changes was assessed using comprehensive pathological scoring, which considered factors including alveolar septal thickening and consolidation, hemorrhage, exudation, pulmonary edema, mucus accumulation, and inflammatory cell recruitment and infiltration in all lung lobes collected from hamsters in each group. The hamsters in groups 1–9 showcased comprehensive lung pathological scores of 10.56, 11.19, 11.13, 8.81, 8.25, 7.94, 3.01, 11.75 and 2.13, respectively (Fig. 5j and Tab S1). Statistic of comprehensive lung pathological scores suggested that hamsters with the combination of three-drug displayed significantly less lung tissue injury than the untreated hamsters and those with single-drug or two-drug therapy (Fig. 5d).

**Fig. 6:**
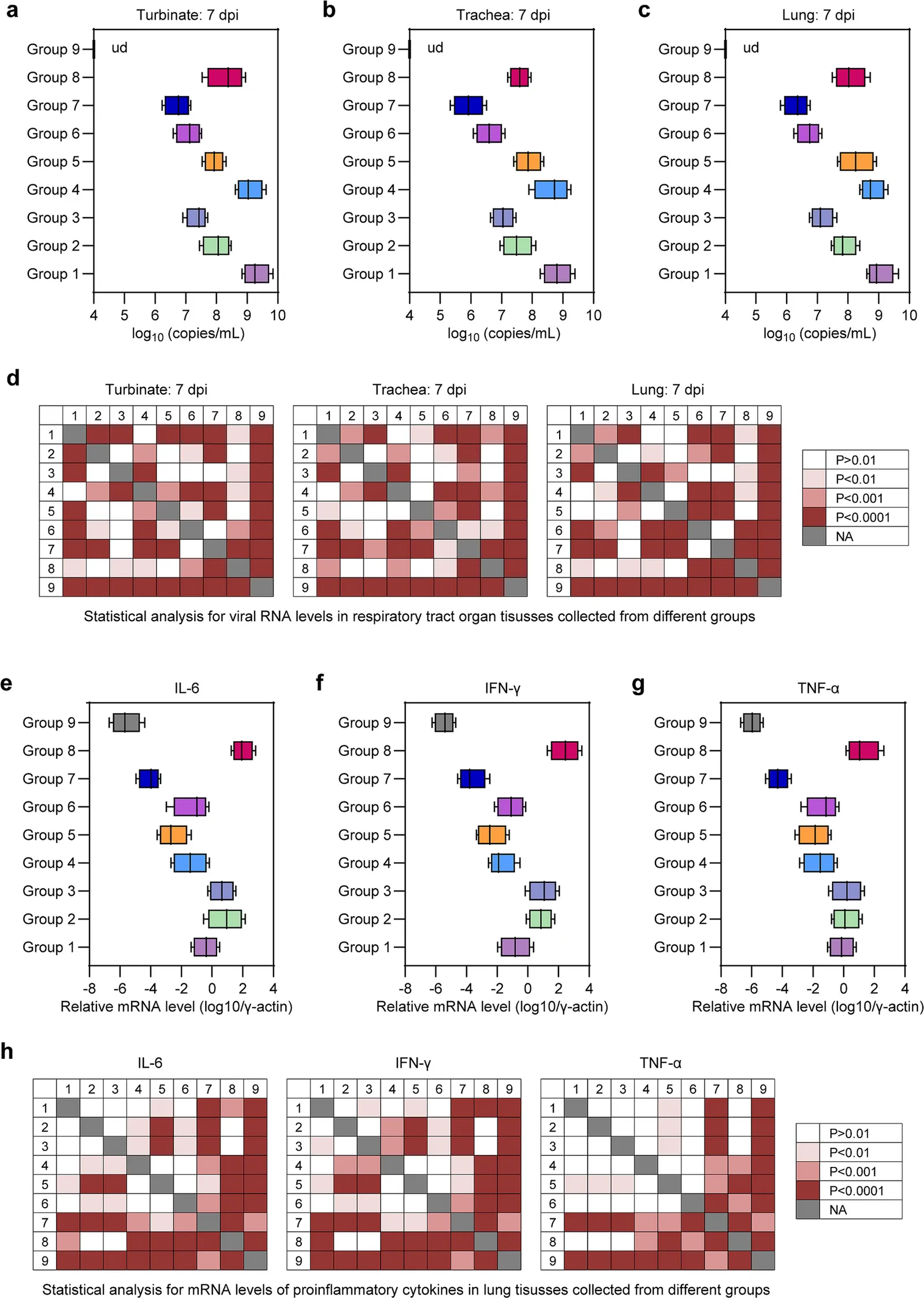
Changes of viral RNA levels and proinflammatory cytokines in respiratory tract organs of the aged hamsters. **a** Nasal turbinate, **b** trachea, and **c** lung tissues of the hamsters in groups 1–9 were collected at 7 dpi. Viral RNA levels in tissue homogenates were measured by qRT-PCR for each group (n = 4). Primers targeting the SARS-CoV-2 ORF1ab gene were used for amplification. **d** Significance of the viral RNA levels was determined by one-way ANOVA and shown in heat map. Two-sided P values < 0.01 were considered significant: *P* < 0.01 (Light red), *P* < 0.001 (Medium red), *P* < 0.0001 (Deep red); *P* > 0.01 indicates no significance (White); “ud” indicates the detected value was under the cut-off level of 1000 copies/mL. The mRNA levels of proinflammatory cytokines, including **e** IL-6, **f** IFN-γ, and **g** TNF-α in the lung tissues of hamsters deceased at 7 dpi were measured by qRT-PCR (n = 4) to determine the fold changes. The mRNA levels were normalized to the house-keeping gene γ-actin. **h** Significance of the mRNA levels was determined by one-way ANOVA and shown in heat map. Two-sided P values < 0.01 were considered significant: *P* < 0.01 (Light red), *P* < 0.001 (Medium red), *P* < 0.0001 (Deep red); *P* > 0.01 indicates no significance (White)

To evaluate viral load, viral RNA loads in the respiratory tract organs (turbinate, trachea, and lung) were quantified by qRT-PCR. The hamsters in groups 1–8 showed viral RNA load of 9.30 ± 0.42, 8.01 ± 0.45, 7.37 ± 0.34, 9.08 ± 0.43, 7.92 ± 0.32, 7.09 ± 0.41, 6.73 ± 0.42 and 8.33 ± 0.61 log_10_ copies/mL in turbinate, respectively (Fig. 6a). The hamsters in groups 1–8 showed viral RNA load of 8.82 ± 0.47, 7.50 ± 0.49, 7.05 ± 0.35, 8.65 ± 0.58, 7.87 ± 0.42, 6.60 ± 0.43, 5.92 ± 0.49 and 7.61 ± 0.32 log_10_ copies/mL in trachea, respectively (Fig. 6b). The hamsters in groups 1–8 showed viral RNA load of 9.03 ± 0.44, 7.88 ± 0.39, 7.14 ± 0.37, 8.80 ± 0.40, 8.27 ± 0.58, 6.72 ± 0.38, 6.31 ± 0.40 and 8.06 ± 0.51 log_10_ copies/mL in lung, respectively (Fig. 6c). Viral RNA was undetectable (below the detection limit) in the respiratory tract organs collected from the mock hamsters in group 9 (Fig. 6a to c). Statistical analysis of viral RNA load suggested that Dex therapy increases viral load and the combination of three-drug showed more efficient inhibitory effect against viral replication in turbinate, trachea and lung than single-drug or two-drug therapy (Fig. 6d). Meanwhile, we evaluated the mRNA levels of proinflammatory cytokines, including IL-6, IFN-γ and TNF-α in lung homogenates collected from hamsters in each group by qRT-PCR, respectively (Fig. 6e to g). Statistical analysis of cytokine mRNA levels suggested that the combination of three-drug showed more potent down-regulation of IL-6, IFN-γ and TNF-α than single-drug or two-drug therapy (Fig. 6h). Overall, these data demonstrate that late intervention with a combination of Dex, Hep and CoVR-MV is an optimal option to suppress viral replication, production of pro-inflammatory cytokines and lung injury after SARS-CoV-2 infection.

To delineate the cross-talk of these pharmacological interactions, we evaluated the combinatorial outcomes using the Bliss independence framework (Fig. S9), which stringently discriminates bona fide therapeutic synergy from mere additive effects. Our synergy landscape revealed that the triple-drug regimen achieved a remarkably high Bliss synergy score in reversing comprehensive pathological damage (61.0, *P* < 0.001). This structural preservation was fundamentally driven by a synergistic collapse of the cytokine storm, as evidenced by the profound cooperative suppression of core inflammatory amplifiers, including IL-6 (27.4, *P* < 0.01) and TNF-α (35.6, *P* < 0.01). Crucially, the Bliss landscape explicitly exposes the inherent limitations of incomplete combinatorial strategies within the aged microenvironment. For instance, the dual combination of dexamethasone and the viral decoy (Dex + CoVR-MV) demonstrated significant pharmacological antagonism regarding viral clearance (TR_RNA: −6.4, *P* < 0.01; Lu_RNA: −9.8, *P* < 0.01). Remarkably, the integration of heparin into this axis (forming the triple therapy) completely abrogated this viral antagonism, restoring virological metrics to a state of positive synergistic clearance (TR_RNA: 12.3, *P* < 0.05). Collectively, these data establish that only the multidimensional approach of the triple therapy can synergistically dismantle the lethal triad of unconstrained viral replication, immune hyperactivation, and microthrombosis in aged hosts. Collectively, the Bliss landscape explicitly proves that the integration of all three agents is pharmacologically indispensable, as only the complete triple regimen can overcome inherent dual-therapy antagonism and synergistically dismantle the lethal pathological triad in aged hosts.

## Discussion

Global deployment of vaccines has significantly reduced the severity of respiratory virus infection and its impact on the adult population [33–35]. However, the high mutation frequency and increasing immune escape ability of the viruses [36, 37], as well as age-associated immune dysfunction and lung function decline, put the elderly population at high risk of respiratory virus infections [17]. As with IFV and RSV, the important understanding of the delicate balance of the interplay between aging and COVID-19 progression that is responsible for the disease outcome of respiratory virus infection is now increasingly appreciated: 1) Robust viral replication and high viral load in respiratory tract organs; 2) Dysfunction of basic physiological functions, complement and coagulation cascades; 3) Excessive activation of cytotoxic NK and T cells, as well as inflammatory signaling and genes; 4) Bidirectional interplay between viral pathology and aging. Because high viral load, immune and coagulation disorders have been demonstrated as main deteriorators of viral pathology [38], achieving a rebalanced immune microenvironment with viral suppression is a crucial route to developing therapeutic strategies against respiratory virus infection in elderly population.

In previous studies, early intervention of Dex or CoVR-MV has been demonstrated to reverse severe pneumonia in critical COVID-19 patients and hamsters with SARS-CoV-2 infection [29, 30, 39–41]. Dex is a steroid drug which has a broad immunosuppressive effect by modulating the abnormal recruitment and proinflammatory differentiation of neutrophils, T lymphocytes and macrophages, as well as inhibiting the process of cytokine storm after infection with SARS-CoV-2 [42–45]. CoVR-MV is a cellular decoy with dual engineering expressions of hACE2 and hDPP4 receptors that mediates a broad direct neutralizing effect against severe acute respiratory syndrome coronavirus (SARS-CoV), Middle East respiratory syndrome coronavirus (MERS-CoV) and SARS-CoV-2. In addition, early intervention with CoVR-MV activates type I interferon signalling in alveolar macrophages and switches the host immune response from inflammatory to antiviral [30]. Hep is a widely used anticoagulant in clinical practice that can inhibit cellular entry of SARS-CoV-2, attenuate COVID-19 symptoms and reduce mortality [46–48]. Despite its anticoagulant properties, Hep has been reported to prevent caspase-11-dependent pyroptosis and death in mouse model [49]. However, the therapeutic effects of late intervention with Dex, Hep, CoVR-MV and their combinations were not clear. The overarching data demonstrated that late intervention of single-drug and two-drug combinations are inadequate to reverse the critical COVID-19 in the aged hamsters. Several disadvantages of single-drug therapy were observed in the animal experiments. Decrease of viral load in respiratory tract organs was not observed in the hamsters with single Hep therapy. Because of its broad immunosuppressive effect, single Dex therapy resulted in more than tenfold increase of viral RNA levels in respiratory tract organs, which delays viral clearance and extends viral shedding. Late intervention of triple-drug combinations can significantly decrease the viral load in the respiratory tract organs and rescue all the aged hamsters from death, sharp loss of body weight, cytokine storm and severe pneumonia. Remarkably, the triple-drug combination compensated for the side-effect of viral load increase in single Dex therapy. Taken together, the synergistic effect of triple-drug combinations is the key point to overcome the challenge of late intervention and age-dependent disease enhancement.

Hamster was widely demonstrated as a highly simulated animal model not only for modeling the disease signature of acute COVID-19 and evaluations of vaccines and drugs, but also for elucidating aging-related COVID-19 pathogenesis, possessing high homology of the ACE2 receptor with its human counterpart and the capacity to naturally recapitulate the progressive deterioration of the pathology immune microenvironment [20, 50–52]. In this study, transcriptomic analysis of lung tissue delineated the features of critical COVID-19 in aged hamsters, and improved our understanding of the pathological mechanisms and complicated interplay between aging and respiratory viral infections. At the pre-infection baseline, our transcriptomic profiling reveals that, compared with adult hamsters, aged hamsters already reside in a microenvironment of chronic inflammaging, consistent with previous reports on the aging human [53]. Following SARS-CoV-2 invasion, the deterioration of targeted immune defenses, specifically encompassing impaired NK cell function and severe naive T cell depletion, precipitates uncontrolled early viral replication. The collapse of specific antiviral immunity forces the host into a compensatory over-reliance on non-specific, highly cytotoxic proinflammatory populations. Such a dysregulated cascade manifests as robust M1 macrophage polarization, extensive neutrophil NETosis, and the aberrant bystander activation of memory T cells, mirroring the immune pathology distinguishing severe human COVID-19 [54]. Crucially, the massive release of neutrophil extracellular traps and potent cytokines inflicts severe endothelial damage and directly activates the local coagulation cascade. The resulting widespread micro-thrombosis severely exacerbates tissue hypoxia and terminal organ failure [55, 56]. Combined with previous physiological, pathological, and immunological research data, the similarity of molecular features between COVID-19 patients and SARS-CoV-2-infected hamsters might improve our understanding of SARS-CoV-2 pathology and inspire new clues of disease interventions and targeted therapy.

Collectively, the explicit transcriptomic mapping of the age-dependent hyperinflammation and immune-thrombosis network establishes a rigorous theoretical foundation for the multidimensional combined therapy targeting viral replication, inflammation, and coagulation. In comparison with previous studies and control groups of single and dual drug therapy, the results of the animal studies revealed the multi-dimensional effects of Dex, Hep and CoVR-MV combined therapy, including collaborative life-saving, viral inhibition, relief of lung injury, decrease of inflammation and coagulation. Furthermore, in ameliorating composite pulmonary pathological damage, the triple combination regimen yielded a substantial synergistic gain of 61.0%. Mechanistically, such profound synergy originates from the simultaneous disruption of the lethal hyperinflammation and immune-thrombosis positive feedback loop. Specifically, CoVR-MV counteracts Dex-induced viral rebound via direct frontline neutralization, whereas Hep bidirectionally cooperates with Dex to jointly suppress inflammation and restore alveolar microcirculation, thereby fundamentally reopening local drug delivery channels to damaged deep tissues [30, 57, 58]. Because severe pneumonia caused by respiratory viruses has a fairly similar pattern of disease progression, we envision that the described strategy of antiviral, anti-coagulation and immune regulation might provide a universal method to defend other respiratory tract viruses with high mutation rates and robust pathogenicity, especially in the elderly population.

Despite these promising findings, our study has certain limitations. First, although the aged Syrian hamster is a robust and highly simulated model for COVID-19, inherent physiological and immunological differences between hamsters and humans may affect the direct clinical translation of our triple-drug regimen. Second, our therapeutic intervention was evaluated at specific late time points; however, human clinical disease courses are highly heterogeneous, requiring further clinical investigation to determine the optimal therapeutic window for aged patients. Future clinical trials are warranted to validate the efficacy and safety of this multi-target strategy in vulnerable elderly cohorts. Because of the lack of antibodies specific to the markers of hamster immune cells, hamster species permissive agents and detection kits, the validation of RNA-Seq results was limited to small-scale FACS analysis of key immune cell subsets and qRT-PCR analysis of the core genes of the pathology network. Collectively, developing relevant technologies and resources is not only urgently needed to expand the application scope and research depth of the hamster model in the field of emerging infectious diseases, but also benefits the fields of immunosenescence, metabolic diseases and cancer treatment.

## Conclusion

In summary, the study reveals that physiological aging fundamentally drives poor disease outcomes in respiratory viral infections, triggering a multifaceted pathogenic network of diffuse cell death, inadequate antiviral responses, excessive inflammation, and immune-thrombosis. Although single- and dual-drug therapies were insufficient to achieve functional cure, the three-drug combination therapy of Dex, Hep, and the viral decoy CoVR-MV successfully rescued the aged hamsters from lethal severe pneumonia through synergistic immunoregulatory, anticoagulant, and antiviral effects. These findings provide valuable clues for the rational design of multi-drug and multi-target therapies against respiratory viral infections and lethal severe pneumonia in the elderly population.

## Materials and methods

### Experimental animal study and sample size

The 10–20-week-old adult male hamsters and 80–90-week-old male hamsters (LVG strain) used in this study were raised in the specific pathogen free animal feeding facilities. To determine the disease outcomes of SARS-CoV-2 infection in adult and aged hamsters, 10 hamsters were set in each group for observation of survival rate within seven days. Body weight data of the survived hamsters from 0 to 7 dpi (including those deceased at 7 dpi) were recorded for evaluation of disease severity (n = 4). Considering the high mortality, lung tissues of mock adult and aged hamsters without infection, and those infected with Delta variant were collected at 5 dpi and fixed in RNAlater for transcriptome analysis in a parallel animal experiment. In an additional parallel animal experiment, the aged and adult hamsters survived from infections of BA.1, BA.5 and XBB.1.16 were observed until 28 dpi (n = 4). For treatment of Delta variant infection in aged hamsters, 10 hamsters were set in each group for observation of survival rate within seven days. Body weight data of the survived hamsters from 0 to 7 dpi (including those deceased at 7 dpi) were recorded for evaluation of disease severity (n = 4). The survived hamsters were euthanized at 7 dpi. Respiratory tract tissues were collected for measurement of viral RNA load, lung injury and inflammation (n = 4). Because all of the hamsters in group 8 were deceased from 0 to 7 dpi, tissue samples collected from those deceased at 7 dpi were used. In an additional parallel animal experiment, the hamsters in groups 1–7 surviving the Delta variant were observed until 28 dpi (n = 4).

### SARS-CoV-2 infection and therapeutic interventions

Hamsters were anesthetized with isoflurane and intranasally inoculated with 1 × 10^4^ plaque-forming units (PFU) of SARS-CoV-2, including the Prototype, Delta, Omicron BA.1, BA.5, and XBB.1.16 variants, respectively. For the therapeutic intervention study against the Delta variant, the infected aged hamsters received either monotherapies or combinations of Dexamethasone (high dose: 1.5, middle dose: 0.5, middle dose: 0.15 mg/kg/day, via intraperitoneal injection), Heparin (high dose: 500, high dose: 500, middle dose: 150, low dose: 50 U/kg/day, via subcutaneous injection), or the virus decoy CoVR-MV (high dose: 1.5, high dose: 0.5, low dose: 0.15 mg/kg/day, via intranasal administration) at 3, 4, and 5 dpi. High dose Nirmatrelvir and Ritonavir treatment scheme (250 nirmatrelvir and 83.3 mg/kg ritonavir, bis in die, from 3 to 5 dpi) was as previously described [59].

### Statistical analysis

All statistical analyses were performed with R software. Survival curves were evaluated using the Kaplan–Meier method and compared by the log-rank test. Continuous variables (e.g., body weight changes, viral RNA loads, and pathological scores) are presented as mean ± SD. For comparisons between two groups, an unpaired Student's t-test was used. For comparisons among multiple groups, a one-way analysis of variance (ANOVA) was applied. Two-sided p-values < 0.01 were considered significant: *P < 0.01, **P < 0.001, ***P < 0.0001, ns indicates no significance.

More detailed information including Biosafety Operations, Virus Inoculation and Sample Collection, Drug Preparation and Regimen, Detection of Viral RNA and Cytokine mRNA, Histopathological Studies and RNA-Seq Data Processing and Analysis was shown in the Supporting Information section.

## Supplementary Information

## Data Availability

The original experimental data are provided as Supplementary Data (Tab S2). Additionally, raw and processed RNA-seq data are available in the NCBI Gene Expression Omnibus (GEO) database (accession: GSE337637). Any further data supporting the findings of this study are available from the corresponding author, Lunzhi Yuan (yuanlunzhi@xmu.edu.cn), upon reasonable request.
