# Supplementary material for: A late-stage multi-dimensional intervention rescues lethal viral pneumonia in an aged hamster model
Source: Mol Biomed. 2026 Sep 2;7:156. doi: 10.1186/s43556-026-00521-5 (PMC13538324; doi:10.1186/s43556-026-00521-5)
Supplement: Supplementary file 1 [file 43556_2026_521_MOESM1_ESM.docx]

A late-stage multi-dimensional intervention rescues lethal viral pneumonia in an aged hamster model

Ming Zhou^1†^, Xuan Liu^2†^, Jinxin Miao^3†^, Yanzhen Lin^4†^, Haiqing Xiao^1†^, Kun Wu^1^, Longmei Zhang^1^, Xinglin Li^1^, Xinyi Yang^1^, Lin Chen^1^, Jian Ma^1^, Wei Wang^1^, Nan Xu^5^, Jiang Li^5^, Huachen Zhu^6,7^, Yi Guan^6,7,8^, Ningshao Xia^1^*, Tong Cheng^1^*, Lunzhi Yuan^1^*

^1^State Key Laboratory of Vaccines for Infectious Diseases, National Institute of Diagnostics and Vaccine Development in Infectious Diseases, Xiang An Biomedicine Laboratory, School of Life Sciences & School of Public Health, Xiamen University, Xiamen, China.

^2^Clinical Center for Biotherapy, Xiamen Key Laboratory of Biotherapy, Clinical Research Center for Precision Medicine of Abdominal Tumor of Fujian Province, Zhongshan Hospital (Xiamen), Fudan University, Xiamen, China.

^3^Academy of Chinese Medicine Science, Henan University of Chinese Medicine, Zhengzhou, China.

^4^Department of Obstetrics and Gynecology, Zhongshan Hospital of Xiamen University, Xiamen, China.

^5^Department of Infectious Diseases, First Affiliated Hospital of Anhui Medical University, Hefei, China.

^6^State Key Laboratory of Emerging Infectious Diseases, School of Public Health, Li Ka Shing Faculty of Medicine, The University of Hong Kong, Hong Kong SAR, China.

^7^Guangdong-Hong Kong Joint Laboratory of Emerging Infectious Diseases, Joint Institute of Virology (STU/HKU), Shantou University, Shantou, China.

^8^Shanghai Institute of Virology, Shanghai-Jiao-Tong University School of Medicine, Shanghai, China.

†Z.M., L.X., M.J.X, L.Y.Z. and X.H.Q. contribute equally

*corresponding authors:

Lunzhi Yuan ([yuanlunzhi@xmu.edu.cn](mailto:yuanlunzhi@xmu.edu.cn))

Tong Cheng ([tcheng@xmu.edu.cn](mailto:tcheng@xmu.edu.cn))

Ningshao Xia ([nsxia@xmu.edu.cn](mailto:nsxia@xmu.edu.cn))

**Biosafety Operations**

All experiments with infectious SARS-CoV-2 were performed in the biosafety level 3 (BSL-3) and animal biosafety level 3 (ABSL-3) facilities. Our staff wore powered air-purifying respirators that filtered the air and disposable coveralls when they cultured the virus and handled animals that were in isolators. The researchers were disinfected before they left the room and then showered on exiting the facility. All facilities, procedures, training records, safety drills, and inventory records were subject to periodic inspections and ongoing oversight by the institutional biosafety officers, who consult frequently with the facility managers.

**Virus Inoculation and Sample Collection**

The SARS-CoV-2 prototype strain AP-8 (EPI_ISL_1655937), as well as the variants including Delta strain AP-1914 (EPI_ISL_2385091), Omicron BA.1 strain AP-309 (EPI_ISL_8182026), BA.5 strain AP-101 (EPI_ISL_12920651) and XBB.1.16 strain AP-144 (EPI_ISL_17684413) were passaged on Vero cells (#CCL-81, ATCC), respectively. Viral stocks were prepared in Vero cells with DMEM containing 2% FBS, 5 ug/mL TPCK-trypsin, 1% Penicillin Streptomycin and 30 mmol/L MgCl_2_. Viruses were harvested and stored in an ultra-low temperature freezer. The titers were determined by means of plaque assay in Vero cells. The 10-20-week-old adult male hamsters and 80-90-week-old male hamsters were anesthetized by isoflurane (#R510-22, RWD Life Science) and nasally inoculated with indicated doses of SARS-CoV-2 diluted in 200 μL of Phosphate Buffered Saline (PBS) (#10010031, GIBCO). In the next seven days, the body weight of these hamsters was measured by an electronic balance.

**Drug Preparation and Regimen**

The preparation and dosage of Dex were adopted from our previous study [1]. Dex (#HY-14648, MCE) was dissolved in DMSO (#D5879, SIGMA-ALDRICH) at a high concentration (20 mg/mL) and stored in an ultra-low temperature refrigerator. The preparation and dosage of Hep were adopted from a previous study of Hep treatment on sepsis. Hep (Enoxaparin Sodium, #S26247, Shanghai-Yuan-Ye) was dissolved in PBS at the concentration of 5000 U/mL and stored in an ultra-low temperature refrigerator. CoVR-MV was generated from hACE2-293T and hDPP4-293T cells within 24 hours prior to use and stored in 4 °C refrigerator as previously reported. The hACE2-293T and hDPP4-293T cells were derived from 293T cell line (#CRL-3216, ATCC) by over-expression of hACE2 or hDPP4 receptor proteins on cell membrane. The cell membrane was collected by a repeated freeze-thaw process. Cell suspensions in PBS were frozen at -80 °C, thawed at room temperature, and pelleted by centrifugation at 1000 g for 5 min. Following three repeated washes with cold PBS mixed with a protease inhibitor cocktail (#87785, Thermo-Scientific), cell membranes were suspended in cold PBS and sonicated in a sterile 1.5 mL EP tube under lower power (22.5 W, 20 s × 3) on ice. Then the vesicles were isolated by multi-step differential centrifugation before being resuspended in PBS. The product was then introduced to a Mini-Extruder (Avanti Polar Lipids) equilibrated in PBS (200 nm pore-sized membrane filters) to further purify uniform nanovesicles. Total membrane protein content was quantified by a BCA protein assay kit (#23227, Thermo-Scientific). For CoVR-MV generation, hACE2-MVs and hDPP4-MVs were mixed and then extruded through 200-nm pores on the Mini-Extruder (Avanti Polar Lipids). For the drug regimen, hamsters were anesthetized with isoflurane.

To maximize systemic bioavailability and closely mimic clinical practices, specific administration routes were selected for each therapeutic agent. Dex stock was dissolved in the volume of 0.2 mL with sesame oil (#S25527, Shanghai-Yuan-Ye) for intraperitoneal (IP) injection. The IP route was utilized to achieve rapid systemic absorption comparable to clinical intravenous (IV) delivery, thereby immediately intercepting the hyper-inflammatory cascade [2]. Hep stock was dissolved in the volume of 0.2 mL with PBS for subcutaneous (SC) injection. As the global clinical gold standard for heparin administration, SC injection ensures a sustained and stable release of the anticoagulant, effectively maintaining the therapeutic window while avoiding the secondary risk of severe hemorrhage associated with IV bolus injections [3]. CoVR-MV was dissolved in the volume of 0.2 mL with PBS for an intranasal regimen. This non-invasive delivery route was specifically designed to achieve maximum local drug concentrations at the respiratory epithelium, directly intercepting viral entry at the primary site of infection and mirroring the physiological infection route of SARS-CoV-2 [4, 5]. The administration route and dose of Nirmatrelvir and Ritonavir were adapted from a previous study [6].

**Detection of Viral RNA and Cytokine mRNA**

For the solid organ samples, we collected 1 mg turbinate, 0.1 mg trachea and 0.1 mg lung in 1 mL PBS for homogenization and detection of viral RNA and viral titer. Viral RNA was extracted using a QIAamp Viral RNA Mini kit (#52906, Qiagen) according to the manufacturer's instructions. The RT-qPCR was conducted using the SLAN-96S Real-Time System (Hongshi, Shanghai, China) with a SARS-CoV-2 RT-qPCR Kit from Wantai (Beijing, China). Relative Viral RNA of SARS-CoV-2 ORF1ab gene was determined using primer pairs and probes provided in the kit. Viral RNA copies were expressed on a log_10_ scale after normalization to the standard curve obtained using ten-fold dilutions of a SARS-CoV-2 stock.

The lung tissues were cleaved into small pieces and soaked in RNAlater (#AM7021, Invitrogen). Total RNAs in lysed lung tissues were extracted with the RNeasy Mini kit (#74106, Qiagen) and reverse-transcribed to cDNA with the Fast-King Strand cDNA Synthesis Kit (#FP313, TIANGEN, Beijing). Diluted cDNAs (1:10) were quantified using SYBR Green I-based qRT-PCR using the Light Cycler® 480 instrument (Roche) per manufacturer’s instructions. Threshold cycle (Ct) of each gene was normalized to the internal reference gene (hamster γ-actin) and the comparative Ct (2^-ΔΔCt^) method was utilized to calculate changes in chemokine and cytokine gene expression profiles. The gene-specific primers (5’ to 3’) used for qRT-PCR were listed in Supplementary Table S2.

**FACS Analysis for the Immune Cells in Hamster Lung Tissues**

Given the limitations of available reagents for the golden hamster, we used antibodies that cross-reacted with divergent species and therefore were likely to recognize the ortholog in hamsters, as described in previous studies [7, 8]. The hamster lung tissues were collected upon perfusion with 30 mL PBS to avoid contamination of lymphocytes from the blood. After collection, lung tissues were dissociated by Miltenyi Gentle MACS with the lung dissociation Kit protocol (#130-095-927). Dissociated lungs were incubated at 37 ℃ for 30 min with 2 mL Collagenase (#17100017, GBICO, 1 mg/mL) and DNase (#D7076, Betotime, 0.5 mg/mL) and passed through 70 μm nylon mesh (FALCON, #352350). For total immune cell-enriched cell count, homogenized lungs were further subjected to Ficoll separation (1200 g, 20 min). For FACS analysis, cells were incubated with a FC block antibody following the staining with indicated surface marker antibodies. For intranuclear staining, cells were fixed for 30 min at 4 ℃ with Fix Buffer I (BD Biosciences) and were then incubated with intracellular marker antibodies overnight at 4 ℃. All samples were incubated with LIVE/DEAD Fixable yellow to exclude dead cells from the analysis. Cells were acquired on a BD LSR Fortessa instrument. The expression of each molecule was assessed with FlowJo software (Tree Star). The anti-CD45 antibody was labeled with Alexa Fluor 594 Antibody Labeling Kit. To analyze the changed proportions of lymphoid and myeloid immune cell subsets, CD4^+^ T follicular helper cell (Tfh) were identified by CD45⁺→CD3⁺→CD4⁺→CXCR5⁺Bcl6⁺; T helper cell 1 (Th1) was identified by CD45⁺→CD3⁺→CD4⁺→CXCR3⁺Foxp3^-^; Naïve CD4^+^ T cell was identified by CD45⁺→CD3⁺→CD4⁺→CD62L^+^CD44^low^; M1-like macrophage was identified by CD45⁺→CD11b⁺→Ly6G⁻→F4/80⁺→CD11c^-^→MHC-II⁺→CD86⁺, respectively. The detailed information of FACS-associated reagents and antibodies was shown in Table S3.

**Histopathological Studies**

For pathological analysis, lung tissues were fixed in formalin for more than 72 hours, dehydrated and then embedded in paraffin wax. The wax blocks of lung tissues was cut into 4 μm sections for pathological staining and analysis. H&E staining was employed for analysis of general lung pathological lesions including pulmonary edema, consolidation, and inflammation. The standards for pathological scores of lung tissues in this study are derived from our previous study in the hamster model. Comprehensive pathological scoring of lung sections was performed according to the degree of lung lesions including alveolar septum hyperplasia, consolidation, and impairment of alveolar structure, fluid exudation, mucus suppository, thrombus, inflammation recruitment, and infiltration of immune cells in each individual lung lobe. For each hamster, three or four lung lobes were employed for evaluation of the comprehensive pathological score. In brief, the H&E staining result of each lung lobe was analyzed for its severity of pathological change. The pathological score included: a) Alveolar septum thickening and consolidation; b) Hemorrhage, exudation, pulmonary edema and mucous; c) Recruitment and infiltration of inflammatory immune cells. For each issue, scores were related to the severity: 0 indicated no pathological change was observed, 1 indicated moderate pathological change, 2 indicated mild pathological change, 3 indicated severe pathological change and 4 indicated very severe pathological change. In conclusion, scores of such three issues were added as the comprehensive pathological score of a lung lobe, and the average comprehensive pathological score of the lobes indicated the severity of lung pathology in an evaluated hamster. The pathological staining reagents include Hematoxylin (#CTS-1096) and Eosin (#CTS-4094), were purchased from Maxim Biotechnology (Fuzhou, China). The images of whole lung lobes were screened by a high-throughput screening microscope system (EVOS M7000, Invitrogen of Thermo-Fisher Scientific).

**Evaluation of Synergistic Effects**

To evaluate the pharmacological interactions of the multi-drug regimens across heterogeneous pathological and molecular metrics, a fractional rescue score was first computed for each sample, standardizing all measurements between the untreated control (defined as 0) and the healthy mock group (defined as 1). Drug synergy was subsequently quantified utilizing the classical Bliss Independence Model, which establishes the theoretical additive expectation of combined therapies assuming mutually independent mechanisms. The final Bliss synergy score was determined by the percentage deviation of the observed combinatorial rescue from the theoretical additive expectation, where positive and negative scores indicated synergistic and antagonistic interactions, respectively. Statistical significance of the synergy scores for each treatment group was assessed via a two-tailed one-sample t-test against a hypothetical mean of zero.

**RNA-Seq Data Processing and Analysis**

Following the assessment of read quality using FastQC, samples that failed to meet the majority of the assessed quality control (QC) statistics were excluded. Low-quality base calls were removed using TrimGalore (v0.6.2; https://github.com/FelixKrueger/TrimGalore). The trimmed reads were then mapped to the reference genome (GCF_017639785.1) with HISAT2 (v2.0.4). This generated SAM files, which were then converted to BAM files by SAMtools (version 1.6; http://www.htslib.org/). Gene expression was quantified using the featureCounts (v1.6.4) software[9], while gene annotations were retrieved with the R package "biomaRt" (v3.6.0). PCA was conducted using the "prcomp" function, following the standardisation of expression values with the "ScaleData" function. The ‘edgeR’ package (v3.28.1) is employed to assess transcriptome alterations resulting from SARS-CoV-2 infection or ageing [10]. For the purpose of transcript change detection, significance was defined as "FDR < 0.01 and |FC| > 2". The false discovery rate was computed with the Benjamini-Hochberg procedure (as implemented in the R function p.adjust) in order to control for type-I error resulting from multiple testing. Subsequently, the "clusterProfiler" package (v4.6.2) is used for statistical analysis of functional profiles for genes with KEGG (https://www.kegg.jp/kegg/) or GO (http://www.geneontology.org/) as a database. Furthermore, the genes were clustered based on their expression using the mfuzz cluster method in the R package ClusterGVis (v0.1.1;https://github.com/junjunlab/ClusterGVis). Each cluster underwent enrichment analysis to functionally categorize it according to the biological processes of GO and KEGG pathways. The relative proportions of distinct immune cell subpopulations were computationally inferred from bulk RNA-sequencing profiles using the CIBERSORT algorithm with 1,000 permutations. Statistical comparisons of cell fractions between experimental groups were performed using the two-sided Wilcoxon rank-sum test. Heatmaps were created with the "pheatmap" package (v1.0.12), while PCA plot and bubble plots were implemented with the "ggplot2" package (v3.3.6) in R language (https://cran.r-project.org/web/packages/ggplot2/index.html).

**Animal and Sample Size Justification**

Sample sizes were maximized considering limits in BSL-3 working capacity, the numbers of animals that can be handled under ABSL-3 conditions and the availability of well-trained staff.

Figure S1. Long-term observation for the aged and adult hamsters surviving infection with BA.1, BA.5 and XBB.1.16 variants of SARS-CoV-2. Serum NAb titers detected by a gradient dilution inhibition assay (left) and body weight changes (right) of the surviving hamsters were evaluated at 14, 21, and 28 dpi (n = 4/group). Significance was determined by one-way ANOVA. **P*<0.01, ***P*<0.001, ****P*<0.0001.

Figure S2. Supplementary transcriptome analysis of hamster lung tissues. The left heatmap illustrates the expression of representative genes that are altered in both adult and old hamsters infected with SARS-CoV-2 in comparison to the mock group. The right heatmap depicts the expression of representative genes that are altered exclusively in older hamsters among those infected with SARS-CoV-2.

Figure S3. Age-dependent profiles of pulmonary immune cell infiltration. Violin plots show the proportions of innate (a) and adaptive (b) immune cell subsets in the lungs of adult and aged hosts under mock or SARS-CoV-2 infection conditions. Colors indicate the experimental groups. Significance was determined by one-way ANOVA. **P* < 0.05, ***P* < 0.01.

Figure S4. FACS analysis for the changes of M1-like macrophage, Naïve CD4^+^ T cell, Th1, and Tfh in the mock hamsters and those infected with SARS-CoV-2. Lung samples were collected from adult and aged hamsters at 5 dpi of Delta variant, as well as indicated mock controls (n = 4/group). Significance was determined by one-way ANOVA. **P* < 0.01, ***P* < 0.001, ****P* < 0.0001.

Figure S5. The mRNA levels for genes of representative signaling components were detected by qRT-PCR. Lung tissues collected from the mock adult and aged hamsters, as well as those infected with Delta variant of SARS-CoV-2 were collected at 5 dpi for qRT-PCR to determine the fold changes of genes associated with severe pneumonia including IL-6, TNF-α, IFN-γ, NLRP3, SERPINE1, Granzyme B, ELANE, MPO, HIF-1α, CCL2, IL-17A and CXCL10, respectively (n = 4/group). The mRNA levels were normalized to the housekeeping gene hamster γ-actin.

Figure S6. Supplementary transcriptome analysis of representative genes of clusters. Heatmap shows genes downregulated after both senescence and SARS-CoV-2 infection compared to the mock group.

Figure S7. Long-term observation of the surviving hamsters in groups 1-7. Serum NAb titers detected by a gradient dilution inhibition assay (left) and body weight changes (right) of the surviving hamsters were evaluated at 14, 21, and 28 dpi (n = 4/group).

Figure S8. Dose-dependent studies of Dex, Hep, CoVR-MV, Nirmatrelvir and Ritonavir. 80-week-old male hamsters were intranasally inoculated with 1×10^4^ PFU of SARS-CoV-2 Delta variant and received drug treatment of different doses at 3, 4, and 5 dpi, respectively. For high dose Nirmatrelvir and Ritonavir treatment, the hamsters received oral administration of 250 mg/kg nirmatrelvir and 83.3 mg/kg ritonavir twice a day. The middle (83.3 mg/kg nirmatrelvir and 27.8 mg/kg ritonavir) and low (27.8 mg/kg nirmatrelvir and 9.3 mg/kg ritonavir) doses were also given in this way. Middle (0.5 mg/kg/day) and low (0.15 mg/kg/day) doses of Dex were given by intraperitoneal injection. Middle (150 U/kg/day) and low (50 U/kg/day) doses of Hep were given by subcutaneous injection. Middle (0.5 mg/kg/day) and low (0.15 mg/kg/day) doses of CoVR-MV were given by intranasal administration. Survival rate of these hamsters was recorded (n = 10/group).

Figure S9. Bliss synergy landscape of the multidimensional combination therapies in aged hosts. Heatmap displaying the Bliss synergy scores of dual- and triple-drug regimens across core pathological, inflammatory, and virological metrics. The synergistic effect was mathematically quantified using the Bliss independence model based on fractional rescue rates. Color gradients represent the degree of interaction: shades of red indicate synergistic effects (Synergy Score > 0), white indicates additive effects (Synergy Score = 0), and shades of blue indicate antagonistic effects (Synergy Score < 0). Significant scores are highlighted in bold. **P* < 0.05, *P* < 0.01, ****P* < 0.001.

**Table S1. Comprehensive pathological scores for the lung lobes.**

| **Group** | **No.** | **Pathological lesions** | | | **Comprehensive pathological score** |
| --- | --- | --- | --- | --- | --- |
|  |  |  |  |  |  |
|  |  | **Alveolar septum hyperplasia and consolidation** | **Pulmonary edema, hemorrhage and mucus suppository** | **Recruitment and infiltration of inflammatory cells** |  |
|  |  |  |  |  |  |
| **Group 1** | **#Group 1-1** | 4+4+4+4 | 4+4+4+4 | 3+3+3+3 | 11+11+11+11 |
|  | **#Group 1-2** | 4+4+4+3 | 4+4+4+3 | 3+3+3+3 | 11+11+11+9 |
|  | **#Group 1-3** | 4+4+4+4 | 4+4+4+4 | 3+3+3+3 | 11+11+11+11 |
|  | **#Group 1-4** | 4+4+3+3 | 4+4+3+2 | 3+3+3+3 | 11+11+9+8 |
| **Group 2** | **#Group 2-1** | 4+4+4+4 | 4+4+4+4 | 3+3+3+3 | 11+11+11+11 |
|  | **#Group 2-2** | 4+4+3+3 | 4+4+3+2 | 4+3+3+2 | 12+11+9+7 |
|  | **#Group 2-3** | 4+4+4+4 | 4+4+4+4 | 4+4+4+4 | 12+12+12+12 |
|  | **#Group 2-4** | 4+4+4+4 | 4+4+4+4 | 4+4+4+4 | 12+12+12+12 |
| **Group 3** | **#Group 3-1** | 4+4+4+4 | 3+3+3+3 | 3+3+3+3 | 10+10+10+10 |
|  | **#Group 3-2** | 4+4+4+3 | 4+4+4+4 | 4+4+4+4 | 12+12+12+11 |
|  | **#Group 3-3** | 4+4+4+4 | 4+4+4+3 | 4+4+3+3 | 12+12+11+10 |
|  | **#Group 3-4** | 4+4+4+3 | 4+4+4+3 | 4+4+4+4 | 12+12+12+10 |
| **Group 4** | **#Group 4-1** | 2+2+2+2 | 2+2+2+2 | 3+2+2+2 | 7+6+6+6 |
|  | **#Group 4-2** | 4+3+2+2 | 4+4+2+2 | 3+3+2+1 | 10+10+6+5 |
|  | **#Group 4-3** | 4+4+4+2 | 4+4+4+2 | 4+4+4+2 | 12+12+12+6 |
|  | **#Group 4-4** | 4+4+3+3 | 4+4+3+3 | 4+4+4+3 | 12+12+10+9 |
| **Group 5** | **#Group 5-1** | 4+3+3+3 | 3+3+3+3 | 4+4+3+3 | 11+11+9+9 |
|  | **#Group 5-2** | 3+2+2+1 | 3+2+1+1 | 4+3+2+1 | 10+7+5+3 |
|  | **#Group 5-3** | 4+3+2+1 | 2+2+2+2 | 4+4+2+2 | 10+9+6+5 |
|  | **#Group 5-4** | 4+3+3+2 | 3+3+2+2 | 4+4+3+3 | 11+11+8+7 |
| **Group 6** | **#Group 6-1** | 3+2+1+1 | 3+2+2+1 | 4+3+1+1 | 10+7+4+3 |
|  | **#Group 6-2** | 4+3+3+1 | 3+3+3+2 | 4+4+4+2 | 11+10+10+5 |
|  | **#Group 6-3** | 4+4+2+0 | 4+4+2+1 | 4+4+2+0 | 12+12+6+1 |
|  | **#Group 6-4** | 4+4+2+2 | 4+4+2+1 | 4+4+3+2 | 12+12+7+5 |
| **Group 7** | **#Group 7-1** | 0+0+0+0 | 1+1+1+1 | 0+0+0+0 | 1+1+1+1 |
|  | **#Group 7-2** | 1+1+1+1 | 1+1+1+1 | 0+0+0+0 | 2+2+2+2 |
|  | **#Group 7-3** | 3+2+2+1 | 2+2+2+1 | 3+2+2+0 | 8+6+6+2 |
|  | **#Group 7-4** | 3+1+0+0 | 2+1+1+1 | 3+2+0+0 | 8+4+1+1 |
| **Group 8** | **#Group 8-1** | 4+4+4+3 | 4+4+4+3 | 4+4+4+4 | 12+12+12+10 |
|  | **#Group 8-2** | 4+4+4+4 | 4+4+4+4 | 4+4+4+4 | 12+12+12+12 |
|  | **#Group 8-3** | 4+4+4+4 | 4+4+4+4 | 4+4+4+4 | 12+12+12+12 |
|  | **#Group 8-4** | 4+4+3+3 | 4+4+4+4 | 4+4+4+4 | 12+12+11+11 |
| **Group 9** | **#Group 9-1** | 1+1+1+1 | 1+1+1+1 | 0+0+0+0 | 2+2+2+2 |
|  | **#Group 9-2** | 1+1+1+1 | 2+2+1+1 | 0+0+0+0 | 3+3+2+2 |
|  | **#Group 9-3** | 1+1+1+1 | 2+1+1+1 | 0+0+0+0 | 3+2+2+2 |
|  | **#Group 9-4** | 2+0+0+0 | 2+1+1+1 | 0+0+0+0 | 4+1+1+1 |

**Table S2. The gene-specific primers (5’ to 3’) used for RT-PCR for hamster specific genes.**

| **Genes** | **Forward** | **Reverse** |
| --- | --- | --- |
| Hamster IFN-γ | TGTTGCTCTGCCTCACTCAGG | AAGACGAGGTCCCCTCCATTC |
| Hamster IL-6 | AGACAAAGCCAGAGTCATT | TCGGTATGCTAAGGCACAG |
| Hamster TNF-α | TGAGCCATCGTGCCAATG | AGCCCGTCTGCTGGTATCAC |
| NLRP3 | TAACTTGCAGAAGCTGGGCTTG | CTCGAAAACCTGATGTGGGGT |
| SERPINE1 | GGCCTCAGAGATTGTGGGTG | GACCGGAAGTTTAGGCCGT |
| Granzyme B | TGCTTCCTTTAAGGGGGACT | TCGTGAAGGCTCTTGGTGTT |
| ELANE | GGCCTCAGAGATTGTGGGTG | GACCGGAAGTTTAGGCCGT |
| MPO | ACTGTAACAACAGACGCAGC | CGTTAGGGAAGCGCACAATG |
| HIF-1α | AGAGCAAAACACACAGCGAA | TGCAGTGAAGCACCTTCCAT |
| CCL2 | GGACCAGAGUCAAGCUAAAUC | UUUAGCUUGACUCUGGUCCAG |
| IL-17A | GGGAACTTCATCTGTGTCTCTG | TGTTGAGGTAGTCTGAGGGC |
| CXCL10 | TCTGAGTGGGACTCAAGGAATC | CAACACGTGGGCAGGATTGA |
| Hamster γ-actin | ACAGAGAGAAGATGACGCAGATAATG | GCCTGA ATGGCCACGTACA |

**Table S3. Reagents for FACS assay.**

| **Reagents** | **Source** | **Identifier** |
| --- | --- | --- |
| FC block antibody (2.4G2) | BD Bioscience | Cat# 553142 |
| Anti-CD45 (HASA25A) | Novus | Cat# NBP2-60909 |
| FITC-anti-CD3 (CD3-12) | BioRad | Cat# MCA1477A488 |
| APC-Cy7-anti-CD4 (GK1.5) | Biolegend | Cat# 100414 |
| Alexa 700-anti-FoxP3 (FJK-16s) | Thermo-Fisher | Cat# 56-5773-82 |
| BV421-anti-CXCR5 (L138D7) | Biolegend | Cat# 145512 |
| PE-anti-Bcl6 (K112-91) | BD Bioscience | Cat# 561522 |
| BV510-anti-CXCR3 (CXCR3-173) | BD Bioscience | Cat# 745033 |
| BV650-anti-CD11b (M1/70) | BD Bioscience | Cat# 563402 |
| PerCP-Cy5.5-anti-Ly-6G (1A8) | BD Bioscience | Cat# 560602 |
| Alexa 488-anti-Ly6C (HK1.4) | BD Bioscience | Cat# 569440 |
| APC-anti-F4/80 (BM8) | Biolegend | Cat# 123116 |
| PE-anti-CD11c (N418) | Biolegend | Cat# 117308 |
| BV605-anti-CD86 (PO3) | Biolegend | Cat# 105125 |
| BV605-anti-CD44 (IM7) | Biolegend | Cat# 103047 |
| PE-anti-CD62L (MEL-14) | Biolegend | Cat# 104407 |
| BV711-anti-Mouse MHC-II (M5/114.15.2) | BD Bioscience | Cat# 563414 |
| Transcription Factor Buffer | BD Biosciences | Cat# 562574 |
| LIVE/DEAD Fixable Yellow | Invitrogen | Cat# L34959 |
| Fix Buffer I | BD Biosciences | Cat# 557870 |
| Perm/Wash Buffer | BD Biosciences | Cat# 557885 |
| Alexa Fluor 594 Antibody Labeling Kit | Invitrogen | Cat# A20185 |

**References for Supporting Information File**

1.	Yuan LZ, Zhou M, Ma J, Liu X, Chen PW, Zhu HC, et al. Dexamethasone ameliorates severe pneumonia but slightly enhances viral replication in the lungs of SARS-CoV-2-infected Syrian hamsters. Cellular & Molecular Immunology. 2022;19(2):290-292. <https://doi.org/10.1038/s41423-021-00793-7>

2.	Turner PV, Brabb T, Pekow C, Vasbinder MA. Administration of substances to laboratory animals: routes of administration and factors to consider. J Am Assoc Lab Anim Sci. 2011;50(5):600-13.

3.	Garcia DA, Baglin TP, Weitz JI, Samama MM. Parenteral anticoagulants: Antithrombotic Therapy and Prevention of Thrombosis, 9th ed: American College of Chest Physicians Evidence-Based Clinical Practice Guidelines. Chest. 2012;141(2 Suppl):e24S-e43S. <https://doi.org/10.1378/chest.11-2291>

4.	Martinez-Delgado G. Inhaled nanobodies against COVID-19. Nat Rev Immunol. 2020;20(10):593. <https://doi.org/10.1038/s41577-020-00443-5>

5.	Liu X, Yuan L, Chen J, Zhang Y, Chen P, Zhou M, et al. Antiviral Nanobiologic Therapy Remodulates Innate Immune Responses to Highly Pathogenic Coronavirus. Advanced science. 2023:e2207249. <https://doi.org/10.1002/advs.202207249>

6.	Cox RM, Lieber CM, Wolf JD, Karimi A, Lieberman NAP, Sticher ZM, et al. Comparing molnupiravir and nirmatrelvir/ritonavir efficacy and the effects on SARS-CoV-2 transmission in animal models. Nat Commun. 2023;14(1):4731. <https://doi.org/10.1038/s41467-023-40556-8>

7.	Horiuchi S, Oishi K, Carrau L, Frere J, Møller R, Panis M, et al. Immune memory from SARS-CoV-2 infection in hamsters provides variant-independent protection but still allows virus transmission. Sci Immunol. 2021;6(66):eabm3131. <https://doi.org/10.1126/sciimmunol.abm3131>

8.	Oishi K, Horiuchi S, Frere J, Schwartz RE, tenOever BR. A diminished immune response underlies age-related SARS-CoV-2 pathologies. Cell Rep. 2022;39(13):111002. <https://doi.org/10.1016/j.celrep.2022.111002>

9.	Liao Y, Smyth GK, Shi W. featureCounts: an efficient general purpose program for assigning sequence reads to genomic features. Bioinformatics. 2014;30(7):923-930. <https://doi.org/10.1093/bioinformatics/btt656>

10.	Robinson MD, McCarthy DJ, Smyth GK. edgeR: a Bioconductor package for differential expression analysis of digital gene expression data. Bioinformatics. 2010;26(1):139-140. <https://doi.org/10.1093/bioinformatics/btp616>
